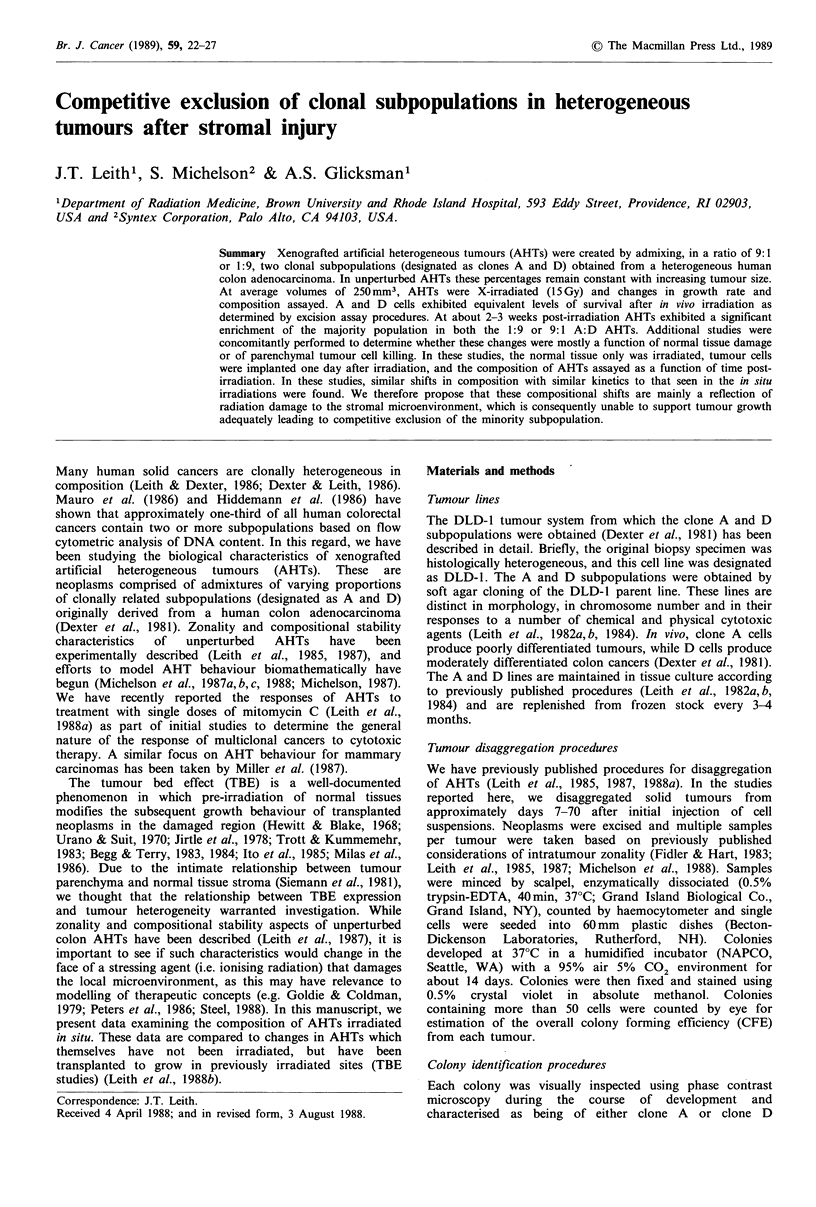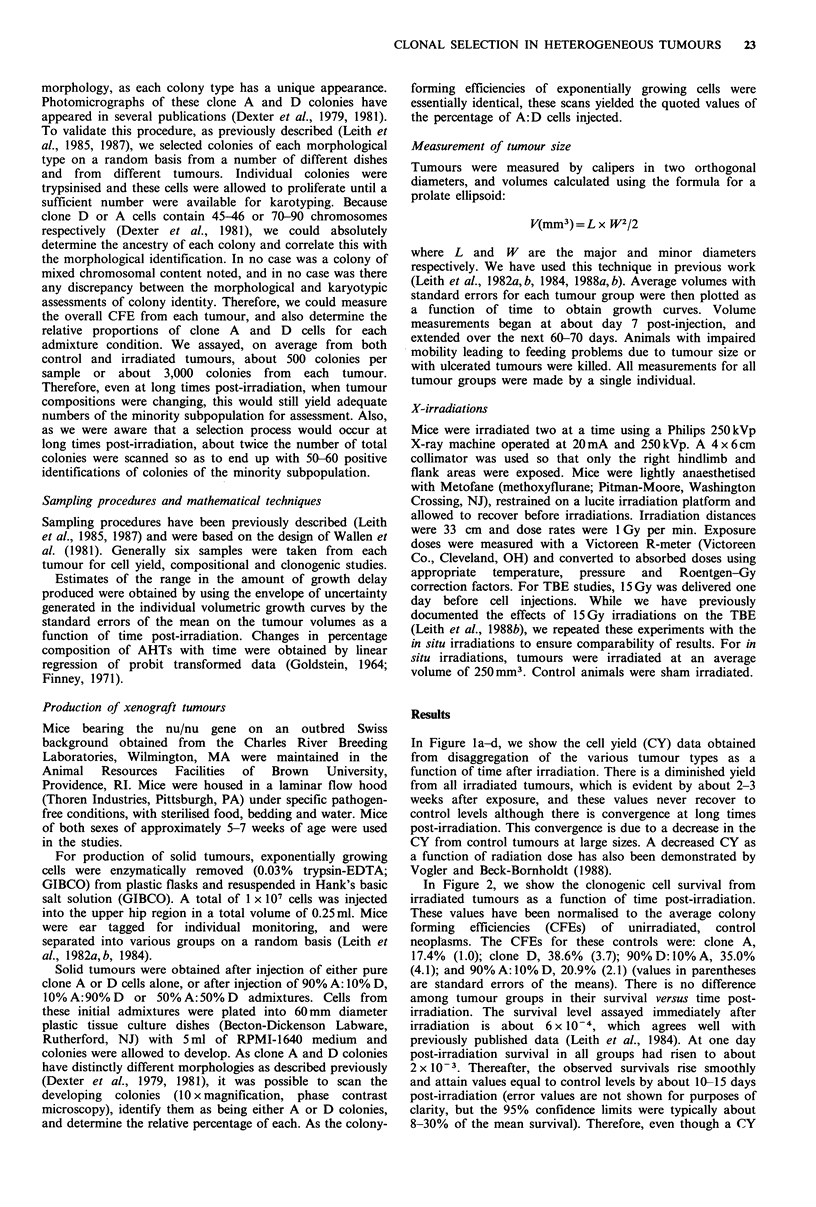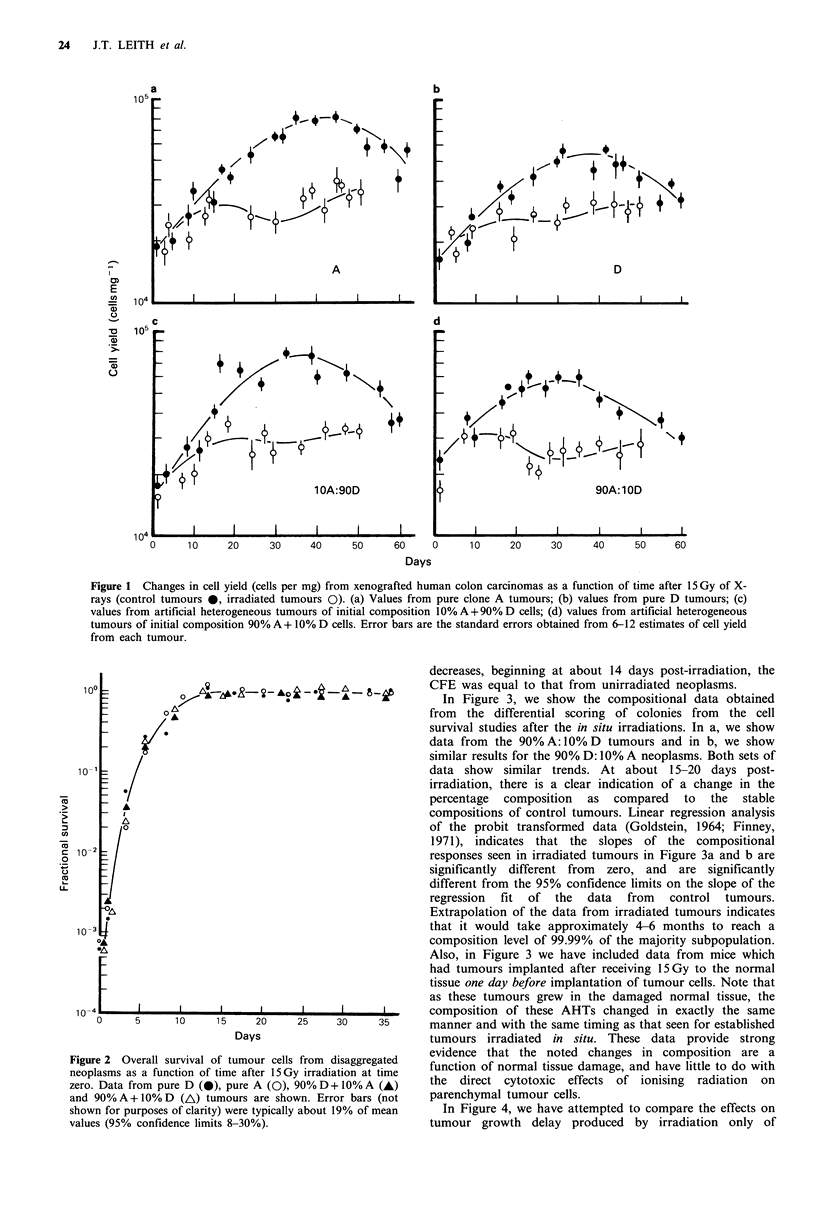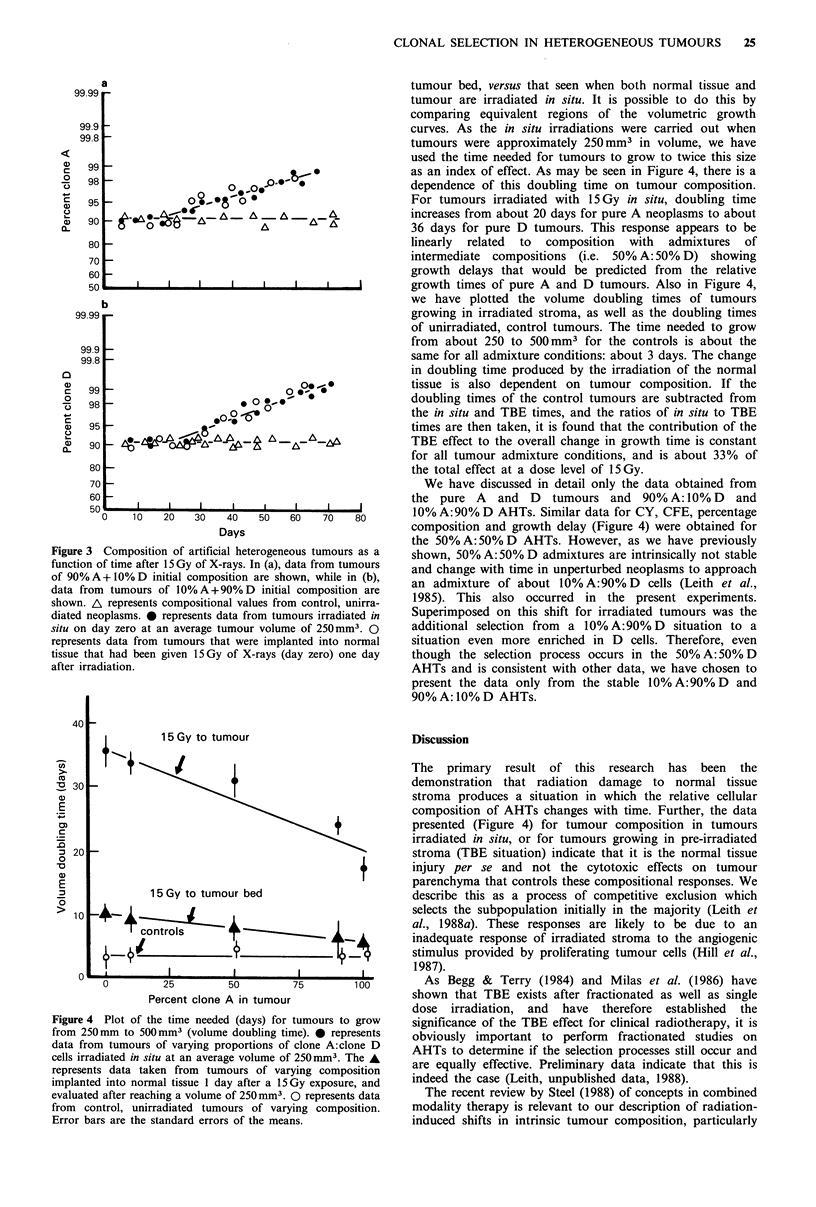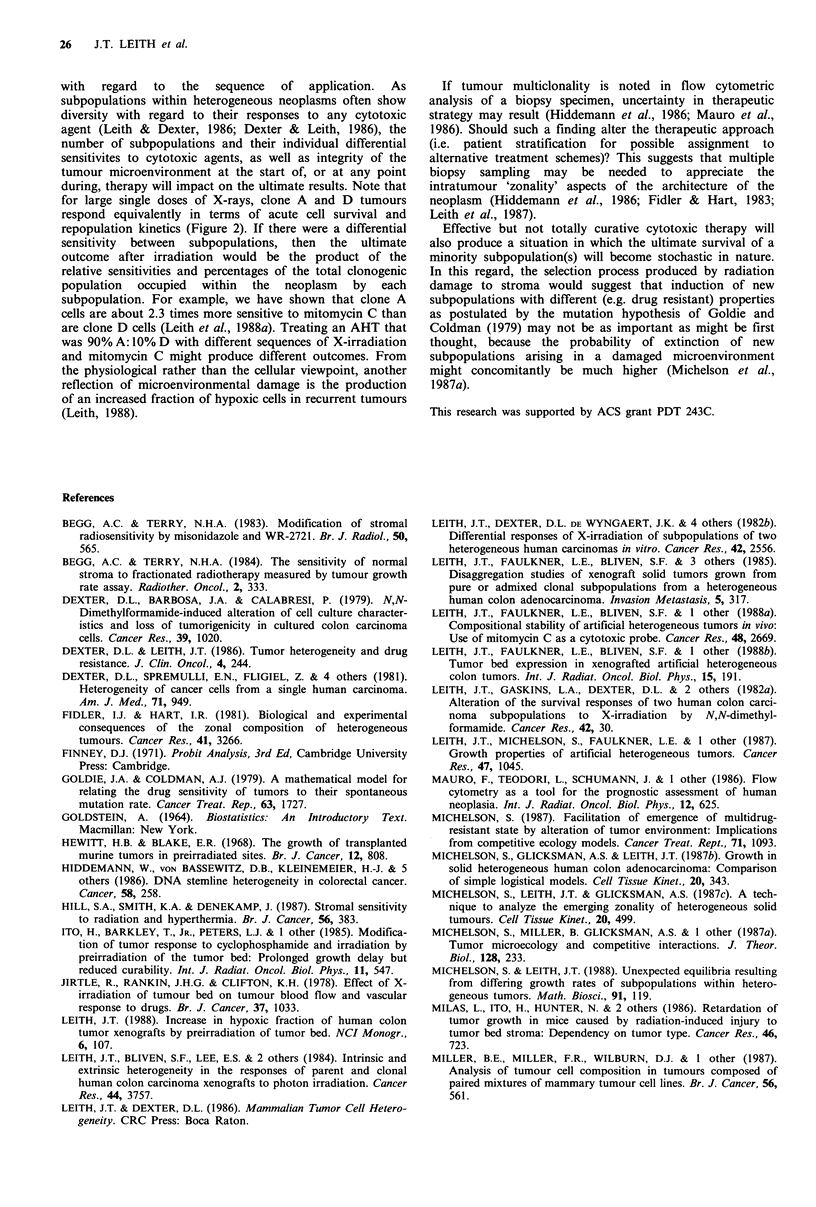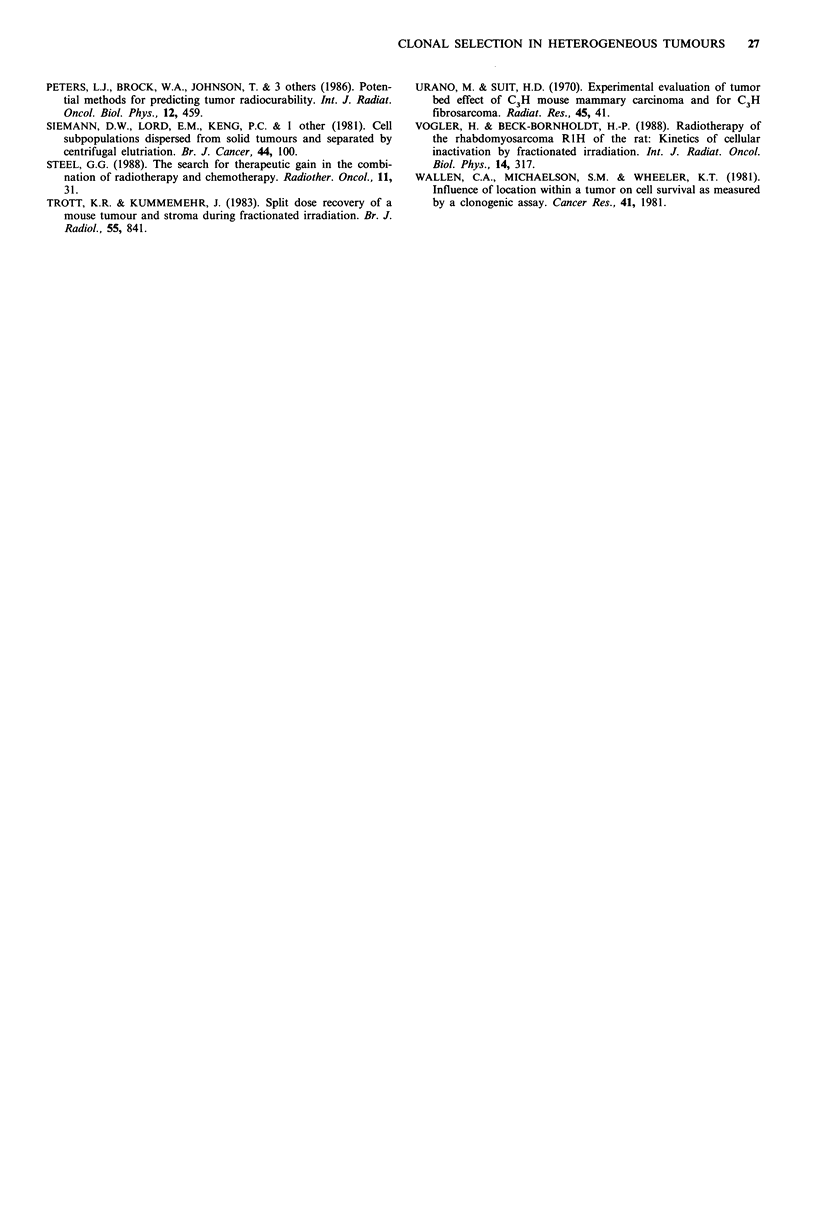# Competitive exclusion of clonal subpopulations in heterogeneous tumours after stromal injury.

**DOI:** 10.1038/bjc.1989.6

**Published:** 1989-01

**Authors:** J. T. Leith, S. Michelson, A. S. Glicksman

**Affiliations:** Department of Radiation Medicine, Brown University, Providence, RI 02903.

## Abstract

Xenografted artificial heterogeneous tumours (AHTs) were created by admixing, in a ratio of 9:1 or 1:9, two clonal subpopulations (designated as clones A and D) obtained from a heterogeneous human colon adenocarcinoma. In unperturbed AHTs these percentages remain constant with increasing tumour size. At average volumes of 250 mm3, AHTs were X-irradiated (15 Gy) and changes in growth rate and composition assayed. A and D cells exhibited equivalent levels of survival after in vivo irradiation as determined by excision assay procedures. At about 2-3 weeks post-irradiation AHTs exhibited a significant enrichment of the majority population in both the 1:9 or 9:1 A:D AHTs. Additional studies were concomitantly performed to determine whether these changes were mostly a function of normal tissue damage or of parenchymal tumour cell killing. In these studies, the normal tissue only was irradiated, tumour cells were implanted one day after irradiation, and the composition of AHTs assayed as a function of time post-irradiation. In these studies, similar shifts in composition with similar kinetics to that seen in the in situ irradiations were found. We therefore propose that these compositional shifts are mainly a reflection of radiation damage to the stromal microenvironment, which is consequently unable to support tumour growth adequately leading to competitive exclusion of the minority subpopulation.


Br. J. Cancer (1989), 59, 22–27 © The Macmillan Press Ltd., 1989

### Competitive exclusion of clonal subpopulations in heterogeneous tumours after stromal injury

J.T. Leith[1], S. Michelson[2] & A.S. Glicksman[1]

[1]*Department of Radiation Medicine, Brown University and Rhode Island Hospital, 593 Eddy Street, Providence, RI 02903, USA and* [2]*Syntex Corporation, Palo Alto, CA 94103, USA.*

**Summary** Xenografted artificial heterogeneous tumours (AHTs) were created by admixing, in a ratio of 9:1 or 1:9, two clonal subpopulations (designated as clones A and D) obtained from a heterogeneous human colon adenocarcinoma. In unperturbed AHTs these percentages remain constant with increasing tumour size. At average volumes of 250 mm$^3$, AHTs were X-irradiated (15 Gy) and changes in growth rate and composition assayed. A and D cells exhibited equivalent levels of survival after *in vivo* irradiation as determined by excision assay procedures. At about 2–3 weeks post-irradiation AHTs exhibited a significant enrichment of the majority population in both the 1:9 or 9:1 A:D AHTs. Additional studies were concomitantly performed to determine whether these changes were mostly a function of normal tissue damage or of parenchymal tumour cell killing. In these studies, the normal tissue only was irradiated, tumour cells were implanted one day after irradiation, and the composition of AHTs assayed as a function of time post-irradiation. In these studies, similar shifts in composition with similar kinetics to that seen in the *in situ* irradiations were found. We therefore propose that these compositional shifts are mainly a reflection of radiation damage to the stromal microenvironment, which is consequently unable to support tumour growth adequately leading to competitive exclusion of the minority subpopulation.

Many human solid cancers are clonally heterogeneous in composition (Leith & Dexter, 1986; Dexter & Leith, 1986). Mauro *et al.* (1986) and Hiddemann *et al.* (1986) have shown that approximately one-third of all human colorectal cancers contain two or more subpopulations based on flow cytometric analysis of DNA content. In this regard, we have been studying the biological characteristics of xenografted artificial heterogeneous tumours (AHTs). These are neoplasms comprised of admixtures of varying proportions of clonally related subpopulations (designated as A and D) originally derived from a human colon adenocarcinoma (Dexter *et al.*, 1981). Zonality and compositional stability characteristics of unperturbed AHTs have been experimentally described (Leith *et al.*, 1985, 1987), and efforts to model AHT behaviour biomathematically have begun (Michelson *et al.*, 1987*a*, *b*, *c*, 1988; Michelson, 1987). We have recently reported the responses of AHTs to treatment with single doses of mitomycin C (Leith *et al.*, 1988*a*) as part of initial studies to determine the general nature of the response of multiclonal cancers to cytotoxic therapy. A similar focus on AHT behaviour for mammary carcinomas has been taken by Miller *et al.* (1987).

The tumour bed effect (TBE) is a well-documented phenomenon in which pre-irradiation of normal tissues modifies the subsequent growth behaviour of transplanted neoplasms in the damaged region (Hewitt & Blake, 1968; Urano & Suit, 1970; Jirtle *et al.*, 1978; Trott & Kummemehr, 1983; Begg & Terry, 1983, 1984; Ito *et al.*, 1985; Milas *et al.*, 1986). Due to the intimate relationship between tumour parenchyma and normal tissue stroma (Siemann *et al.*, 1981), we thought that the relationship between TBE expression and tumour heterogeneity warranted investigation. While zonality and compositional stability aspects of unperturbed colon AHTs have been described (Leith *et al.*, 1987), it is important to see if such characteristics would change in the face of a stressing agent (i.e. ionising radiation) that damages the local microenvironment, as this may have relevance to modelling of therapeutic concepts (e.g. Goldie & Coldman, 1979; Peters *et al.*, 1986; Steel, 1988). In this manuscript, we present data examining the composition of AHTs irradiated *in situ*. These data are compared to changes in AHTs which themselves have not been irradiated, but have been transplanted to grow in previously irradiated sites (TBE studies) (Leith *et al.*, 1988*b*).

Correspondence: J.T. Leith.
Received 4 April 1988; and in revised form, 3 August 1988.

### Materials and methods

### *Tumour lines*

The DLD-1 tumour system from which the clone A and D subpopulations were obtained (Dexter *et al.*, 1981) has been described in detail. Briefly, the original biopsy specimen was histologically heterogeneous, and this cell line was designated as DLD-1. The A and D subpopulations were obtained by soft agar cloning of the DLD-1 parent line. These lines are distinct in morphology, in chromosome number and in their responses to a number of chemical and physical cytotoxic agents (Leith *et al.*, 1982*a*, *b*, 1984). *In vivo*, clone A cells produce poorly differentiated tumours, while D cells produce moderately differentiated colon cancers (Dexter *et al.*, 1981). The A and D lines are maintained in tissue culture according to previously published procedures (Leith *et al.*, 1982*a*, *b*, 1984) and are replenished from frozen stock every 3–4 months.

### *Tumour disaggregation procedures*

We have previously published procedures for disaggregation of AHTs (Leith *et al.*, 1985, 1987, 1988*a*). In the studies reported here, we disaggregated solid tumours from approximately days 7–70 after initial injection of cell suspensions. Neoplasms were excised and multiple samples per tumour were taken based on previously published considerations of intratumour zonality (Fidler & Hart, 1983; Leith *et al.*, 1985, 1987; Michelson *et al.*, 1988). Samples were minced by scalpel, enzymatically dissociated (0.5% trypsin-EDTA, 40 min, 37°C; Grand Island Biological Co., Grand Island, NY), counted by haemocytometer and single cells were seeded into 60 mm plastic dishes (Becton-Dickenson Laboratories, Rutherford, NH). Colonies developed at 37°C in a humidified incubator (NAPCO, Seattle, WA) with a 95% air 5% $CO_2$ environment for about 14 days. Colonies were then fixed and stained using 0.5% crystal violet in absolute methanol. Colonies containing more than 50 cells were counted by eye for estimation of the overall colony forming efficiency (CFE) from each tumour.

### *Colony identification procedures*

Each colony was visually inspected using phase contrast microscopy during the course of development and characterised as being of either clone A or clone D

morphology, as each colony type has a unique appearance. Photomicrographs of these clone A and D colonies have appeared in several publications (Dexter *et al.*, 1979, 1981). To validate this procedure, as previously described (Leith *et al.*, 1985, 1987), we selected colonies of each morphological type on a random basis from a number of different dishes and from different tumours. Individual colonies were trypsinised and these cells were allowed to proliferate until a sufficient number were available for karotyping. Because clone D or A cells contain 45–46 or 70–90 chromosomes respectively (Dexter *et al.*, 1981), we could absolutely determine the ancestry of each colony and correlate this with the morphological identification. In no case was a colony of mixed chromosomal content noted, and in no case was there any discrepancy between the morphological and karyotypic assessments of colony identity. Therefore, we could measure the overall CFE from each tumour, and also determine the relative proportions of clone A and D cells for each admixture condition. We assayed, on average from both control and irradiated tumours, about 500 colonies per sample or about 3,000 colonies from each tumour. Therefore, even at long times post-irradiation, when tumour compositions were changing, this would still yield adequate numbers of the minority subpopulation for assessment. Also, as we were aware that a selection process would occur at long times post-irradiation, about twice the number of total colonies were scanned so as to end up with 50–60 positive identifications of colonies of the minority subpopulation.

*Sampling procedures and mathematical techniques*

Sampling procedures have been previously described (Leith *et al.*, 1985, 1987) and were based on the design of Wallen *et al.* (1981). Generally six samples were taken from each tumour for cell yield, compositional and clonogenic studies.

Estimates of the range in the amount of growth delay produced were obtained by using the envelope of uncertainty generated in the individual volumetric growth curves by the standard errors of the mean on the tumour volumes as a function of time post-irradiation. Changes in percentage composition of AHTs with time were obtained by linear regression of probit transformed data (Goldstein, 1964; Finney, 1971).

*Production of xenograft tumours*

Mice bearing the nu/nu gene on an outbred Swiss background obtained from the Charles River Breeding Laboratories, Wilmington, MA were maintained in the Animal Resources Facilities of Brown University, Providence, RI. Mice were housed in a laminar flow hood (Thoren Industries, Pittsburgh, PA) under specific pathogen-free conditions, with sterilised food, bedding and water. Mice of both sexes of approximately 5–7 weeks of age were used in the studies.

For production of solid tumours, exponentially growing cells were enzymatically removed (0.03% trypsin-EDTA; GIBCO) from plastic flasks and resuspended in Hank's basic salt solution (GIBCO). A total of $1 \times 10^7$ cells was injected into the upper hip region in a total volume of 0.25 ml. Mice were ear tagged for individual monitoring, and were separated into various groups on a random basis (Leith *et al.*, 1982*a*,*b*, 1984).

Solid tumours were obtained after injection of either pure clone A or D cells alone, or after injection of 90% A:10% D, 10% A:90% D or 50% A:50% D admixtures. Cells from these initial admixtures were plated into 60 mm diameter plastic tissue culture dishes (Becton-Dickenson Labware, Rutherford, NJ) with 5 ml of RPMI-1640 medium and colonies were allowed to develop. As clone A and D colonies have distinctly different morphologies as described previously (Dexter *et al.*, 1979, 1981), it was possible to scan the developing colonies (10 × magnification, phase contrast microscopy), identify them as being either A or D colonies, and determine the relative percentage of each. As the colony-forming efficiencies of exponentially growing cells were essentially identical, these scans yielded the quoted values of the percentage of A:D cells injected.

*Measurement of tumour size*

Tumours were measured by calipers in two orthogonal diameters, and volumes calculated using the formula for a prolate ellipsoid:

$$V(\text{mm}^3) = L \times W^2/2$$

where $L$ and $W$ are the major and minor diameters respectively. We have used this technique in previous work (Leith *et al.*, 1982*a*,*b*, 1984, 1988*a*,*b*). Average volumes with standard errors for each tumour group were then plotted as a function of time to obtain growth curves. Volume measurements began at about day 7 post-injection, and extended over the next 60–70 days. Animals with impaired mobility leading to feeding problems due to tumour size or with ulcerated tumours were killed. All measurements for all tumour groups were made by a single individual.

*X-irradiations*

Mice were irradiated two at a time using a Philips 250 kVp X-ray machine operated at 20 mA and 250 kVp. A 4 × 6 cm collimator was used so that only the right hindlimb and flank areas were exposed. Mice were lightly anaesthetised with Metofane (methoxyflurane; Pitman-Moore, Washington Crossing, NJ), restrained on a lucite irradiation platform and allowed to recover before irradiations. Irradiation distances were 33 cm and dose rates were 1 Gy per min. Exposure doses were measured with a Victoreen R-meter (Victoreen Co., Cleveland, OH) and converted to absorbed doses using appropriate temperature, pressure and Roentgen–Gy correction factors. For TBE studies, 15 Gy was delivered one day before cell injections. While we have previously documented the effects of 15 Gy irradiations on the TBE (Leith *et al.*, 1988*b*), we repeated these experiments with the *in situ* irradiations to ensure comparability of results. For *in situ* irradiations, tumours were irradiated at an average volume of 250 mm³. Control animals were sham irradiated.

### Results

In Figure 1a–d, we show the cell yield (CY) data obtained from disaggregation of the various tumour types as a function of time after irradiation. There is a diminished yield from all irradiated tumours, which is evident by about 2–3 weeks after exposure, and these values never recover to control levels although there is convergence at long times post-irradiation. This convergence is due to a decrease in the CY from control tumours at large sizes. A decreased CY as a function of radiation dose has also been demonstrated by Vogler and Beck-Bornholdt (1988).

In Figure 2, we show the clonogenic cell survival from irradiated tumours as a function of time post-irradiation. These values have been normalised to the average colony forming efficiencies (CFEs) of unirradiated, control neoplasms. The CFEs for these controls were: clone A, 17.4% (1.0); clone D, 38.6% (3.7); 90% D:10% A, 35.0% (4.1); and 90% A:10% D, 20.9% (2.1) (values in parentheses are standard errors of the means). There is no difference among tumour groups in their survival *versus* time post-irradiation. The survival level assayed immediately after irradiation is about $6 \times 10^{-4}$, which agrees well with previously published data (Leith *et al.*, 1984). At one day post-irradiation survival in all groups had risen to about $2 \times 10^{-3}$. Thereafter, the observed survivals rise smoothly and attain values equal to control levels by about 10–15 days post-irradiation (error values are not shown for purposes of clarity, but the 95% confidence limits were typically about 8–30% of the mean survival). Therefore, even though a CY

**Figure 1** Changes in cell yield (cells per mg) from xenografted human colon carcinomas as a function of time after 15 Gy of X-rays (control tumours ●, irradiated tumours ○). (a) Values from pure clone A tumours; (b) values from pure D tumours; (c) values from artificial heterogeneous tumours of initial composition 10% A + 90% D cells; (d) values from artificial heterogeneous tumours of initial composition 90% A + 10% D cells. Error bars are the standard errors obtained from 6–12 estimates of cell yield from each tumour.

**Figure 2** Overall survival of tumour cells from disaggregated neoplasms as a function of time after 15 Gy irradiation at time zero. Data from pure D (●), pure A (○), 90% D + 10% A (▲) and 90% A + 10% D (△) tumours are shown. Error bars (not shown for purposes of clarity) were typically about 19% of mean values (95% confidence limits 8–30%).

decreases, beginning at about 14 days post-irradiation, the CFE was equal to that from unirradiated neoplasms.

In Figure 3, we show the compositional data obtained from the differential scoring of colonies from the cell survival studies after the *in situ* irradiations. In a, we show data from the 90% A:10% D tumours and in b, we show similar results for the 90% D:10% A neoplasms. Both sets of data show similar trends. At about 15–20 days post-irradiation, there is a clear indication of a change in the percentage composition as compared to the stable compositions of control tumours. Linear regression analysis of the probit transformed data (Goldstein, 1964; Finney, 1971), indicates that the slopes of the compositional responses seen in irradiated tumours in Figure 3a and b are significantly different from zero, and are significantly different from the 95% confidence limits on the slope of the regression fit of the data from control tumours. Extrapolation of the data from irradiated tumours indicates that it would take approximately 4–6 months to reach a composition level of 99.99% of the majority subpopulation. Also, in Figure 3 we have included data from mice which had tumours implanted after receiving 15 Gy to the normal tissue *one day before* implantation of tumour cells. Note that as these tumours grew in the damaged normal tissue, the composition of these AHTs changed in exactly the same manner and with the same timing as that seen for established tumours irradiated *in situ*. These data provide strong evidence that the noted changes in composition are a function of normal tissue damage, and have little to do with the direct cytotoxic effects of ionising radiation on parenchymal tumour cells.

In Figure 4, we have attempted to compare the effects on tumour growth delay produced by irradiation only of

**Figure 3** Composition of artificial heterogeneous tumours as a function of time after 15 Gy of X-rays. In (a), data from tumours of 90% A + 10% D initial composition are shown, while in (b), data from tumours of 10% A + 90% D initial composition are shown. △ represents compositional values from control, unirradiated neoplasms. ● represents data from tumours irradiated *in situ* on day zero at an average tumour volume of 250 mm³. ○ represents data from tumours that were implanted into normal tissue that had been given 15 Gy of X-rays (day zero) one day after irradiation.

**Figure 4** Plot of the time needed (days) for tumours to grow from 250 mm to 500 mm³ (volume doubling time). ● represents data from tumours of varying proportions of clone A:clone D cells irradiated *in situ* at an average volume of 250 mm³. The ▲ represents data taken from tumours of varying composition implanted into normal tissue 1 day after a 15 Gy exposure, and evaluated after reaching a volume of 250 mm³. ○ represents data from control, unirradiated tumours of varying composition. Error bars are the standard errors of the means.

tumour bed, *versus* that seen when both normal tissue and tumour are irradiated *in situ*. It is possible to do this by comparing equivalent regions of the volumetric growth curves. As the *in situ* irradiations were carried out when tumours were approximately 250 mm³ in volume, we have used the time needed for tumours to grow to twice this size as an index of effect. As may be seen in Figure 4, there is a dependence of this doubling time on tumour composition. For tumours irradiated with 15 Gy *in situ*, doubling time increases from about 20 days for pure A neoplasms to about 36 days for pure D tumours. This response appears to be linearly related to composition with admixtures of intermediate compositions (i.e. 50% A:50% D) showing growth delays that would be predicted from the relative growth times of pure A and D tumours. Also in Figure 4, we have plotted the volume doubling times of tumours growing in irradiated stroma, as well as the doubling times of unirradiated, control tumours. The time needed to grow from about 250 to 500 mm³ for the controls is about the same for all admixture conditions: about 3 days. The change in doubling time produced by the irradiation of the normal tissue is also dependent on tumour composition. If the doubling times of the control tumours are subtracted from the *in situ* and TBE times, and the ratios of *in situ* to TBE times are then taken, it is found that the contribution of the TBE effect to the overall change in growth time is constant for all tumour admixture conditions, and is about 33% of the total effect at a dose level of 15 Gy.

We have discussed in detail only the data obtained from the pure A and D tumours and 90% A:10% D and 10% A:90% D AHTs. Similar data for CY, CFE, percentage composition and growth delay (Figure 4) were obtained for the 50% A:50% D AHTs. However, as we have previously shown, 50% A:50% D admixtures are intrinsically not stable and change with time in unperturbed neoplasms to approach an admixture of about 10% A:90% D cells (Leith *et al.*, 1985). This also occurred in the present experiments. Superimposed on this shift for irradiated tumours was the additional selection from a 10% A:90% D situation to a situation even more enriched in D cells. Therefore, even though the selection process occurs in the 50% A:50% D AHTs and is consistent with other data, we have chosen to present the data only from the stable 10% A:90% D and 90% A:10% D AHTs.

### Discussion

The primary result of this research has been the demonstration that radiation damage to normal tissue stroma produces a situation in which the relative cellular composition of AHTs changes with time. Further, the data presented (Figure 4) for tumour composition in tumours irradiated *in situ*, or for tumours growing in pre-irradiated stroma (TBE situation) indicate that it is the normal tissue injury *per se* and not the cytotoxic effects on tumour parenchyma that controls these compositional responses. We describe this as a process of competitive exclusion which selects the subpopulation initially in the majority (Leith *et al.*, 1988*a*). These responses are likely to be due to an inadequate response of irradiated stroma to the angiogenic stimulus provided by proliferating tumour cells (Hill *et al.*, 1987).

As Begg & Terry (1984) and Milas *et al.* (1986) have shown that TBE exists after fractionated as well as single dose irradiation, and have therefore established the significance of the TBE effect for clinical radiotherapy, it is obviously important to perform fractionated studies on AHTs to determine if the selection processes still occur and are equally effective. Preliminary data indicate that this is indeed the case (Leith, unpublished data, 1988).

The recent review by Steel (1988) of concepts in combined modality therapy is relevant to our description of radiation-induced shifts in intrinsic tumour composition, particularly with regard to the sequence of application. As subpopulations within heterogeneous neoplasms often show diversity with regard to their responses to any cytotoxic agent (Leith & Dexter, 1986; Dexter & Leith, 1986), the number of subpopulations and their individual differential sensitivites to cytotoxic agents, as well as integrity of the tumour microenvironment at the start of, or at any point during, therapy will impact on the ultimate results. Note that for large single doses of X-rays, clone A and D tumours respond equivalently in terms of acute cell survival and repopulation kinetics (Figure 2). If there were a differential sensitivity between subpopulations, then the ultimate outcome after irradiation would be the product of the relative sensitivities and percentages of the total clonogenic population occupied within the neoplasm by each subpopulation. For example, we have shown that clone A cells are about 2.3 times more sensitive to mitomycin C than are clone D cells (Leith *et al.*, 1988*a*). Treating an AHT that was 90% A:10% D with different sequences of X-irradiation and mitomycin C might produce different outcomes. From the physiological rather than the cellular viewpoint, another reflection of microenvironmental damage is the production of an increased fraction of hypoxic cells in recurrent tumours (Leith, 1988).

If tumour multiclonality is noted in flow cytometric analysis of a biopsy specimen, uncertainty in therapeutic strategy may result (Hiddemann *et al.*, 1986; Mauro *et al.*, 1986). Should such a finding alter the therapeutic approach (i.e. patient stratification for possible assignment to alternative treatment schemes)? This suggests that multiple biopsy sampling may be needed to appreciate the intratumour 'zonality' aspects of the architecture of the neoplasm (Hiddemann *et al.*, 1986; Fidler & Hart, 1983; Leith *et al.*, 1987).

Effective but not totally curative cytotoxic therapy will also produce a situation in which the ultimate survival of a minority subpopulation(s) will become stochastic in nature. In this regard, the selection process produced by radiation damage to stroma would suggest that induction of new subpopulations with different (e.g. drug resistant) properties as postulated by the mutation hypothesis of Goldie and Coldman (1979) may not be as important as might be first thought, because the probability of extinction of new subpopulations arising in a damaged microenvironment might concomitantly be much higher (Michelson *et al.*, 1987*a*).

This research was supported by ACS grant PDT 243C.

### References

BEGG, A.C. & TERRY, N.H.A. (1983). Modification of stromal radiosensitivity by misonidazole and WR-2721. *Br. J. Radiol.*, **50**, 565.

BEGG, A.C. & TERRY, N.H.A. (1984). The sensitivity of normal stroma to fractionated radiotherapy measured by tumour growth rate assay. *Radiother. Oncol.*, **2**, 333.

DEXTER, D.L., BARBOSA, J.A. & CALABRESI, P. (1979). *N,N*-Dimethylformamide-induced alteration of cell culture characteristics and loss of tumorigenicity in cultured colon carcinoma cells. *Cancer Res.*, **39**, 1020.

DEXTER, D.L. & LEITH, J.T. (1986). Tumor heterogeneity and drug resistance. *J. Clin. Oncol.*, **4**, 244.

DEXTER, D.L., SPREMULLI, E.N., FLIGIEL, Z. & 4 others (1981). Heterogeneity of cancer cells from a single human carcinoma. *Am. J. Med.*, **71**, 949.

FIDLER, I.J. & HART, I.R. (1981). Biological and experimental consequences of the zonal composition of heterogeneous tumours. *Cancer Res.*, **41**, 3266.

FINNEY, D.J. (1971). *Probit Analysis, 3rd Ed*, Cambridge University Press: Cambridge.

GOLDIE, J.A. & COLDMAN, A.J. (1979). A mathematical model for relating the drug sensitivity of tumors to their spontaneous mutation rate. *Cancer Treat. Rep.*, **63**, 1727.

GOLDSTEIN, A. (1964). *Biostatistics: An Introductory Text.* Macmillan: New York.

HEWITT, H.B. & BLAKE, E.R. (1968). The growth of transplanted murine tumors in preirradiated sites. *Br. J. Cancer*, **12**, 808.

HIDDEMANN, W., von BASSEWITZ, D.B., KLEINEMEIER, H.-J. & 5 others (1986). DNA stemline heterogeneity in colorectal cancer. *Cancer*, **58**, 258.

HILL, S.A., SMITH, K.A. & DENEKAMP, J. (1987). Stromal sensitivity to radiation and hyperthermia. *Br. J. Cancer*, **56**, 383.

ITO, H., BARKLEY, T., Jr., PETERS, L.J. & 1 other (1985). Modification of tumor response to cyclophosphamide and irradiation by preirradiation of the tumor bed: Prolonged growth delay but reduced curability. *Int. J. Radiat. Oncol. Biol. Phys.*, **11**, 547.

JIRTLE, R., RANKIN, J.H.G. & CLIFTON, K.H. (1978). Effect of X-irradiation of tumour bed on tumour blood flow and vascular response to drugs. *Br. J. Cancer*, **37**, 1033.

LEITH, J.T. (1988). Increase in hypoxic fraction of human colon tumor xenografts by preirradiation of tumor bed. *NCI Monogr.*, **6**, 107.

LEITH, J.T., BLIVEN, S.F., LEE, E.S. & 2 others (1984). Intrinsic and extrinsic heterogeneity in the responses of parent and clonal human colon carcinoma xenografts to photon irradiation. *Cancer Res.*, **44**, 3757.

LEITH, J.T. & DEXTER, D.L. (1986). *Mammalian Tumor Cell Heterogeneity.* CRC Press: Boca Raton.

LEITH, J.T., DEXTER, D.L. de WYNGAERT, J.K. & 4 others (1982*b*). Differential responses of X-irradiation of subpopulations of two heterogeneous human carcinomas *in vitro*. *Cancer Res.*, **42**, 2556.

LEITH, J.T., FAULKNER, L.E., BLIVEN, S.F. & 3 others (1985). Disaggregation studies of xenograft solid tumors grown from pure or admixed clonal subpopulations from a heterogeneous human colon adenocarcinoma. *Invasion Metastasis*, **5**, 317.

LEITH, J.T., FAULKNER, L.E., BLIVEN, S.F. & 1 other (1988*a*). Compositional stability of artificial heterogeneous tumors *in vivo*: Use of mitomycin C as a cytotoxic probe. *Cancer Res.*, **48**, 2669.

LEITH, J.T., FAULKNER, L.E., BLIVEN, S.F. & 1 other (1988*b*). Tumor bed expression in xenografted artificial heterogeneous colon tumors. *Int. J. Radiat. Oncol. Biol. Phys.*, **15**, 191.

LEITH, J.T., GASKINS, L.A., DEXTER, D.L. & 2 others (1982*a*). Alteration of the survival responses of two human colon carcinoma subpopulations to X-irradiation by *N,N*-dimethylformamide. *Cancer Res.*, **42**, 30.

LEITH, J.T., MICHELSON, S., FAULKNER, L.E. & 1 other (1987). Growth properties of artificial heterogeneous tumors. *Cancer Res.*, **47**, 1045.

MAURO, F., TEODORI, L., SCHUMANN, J. & 1 other (1986). Flow cytometry as a tool for the prognostic assessment of human neoplasia. *Int. J. Radiat. Oncol. Biol. Phys.*, **12**, 625.

MICHELSON, S. (1987). Facilitation of emergence of multidrug-resistant state by alteration of tumor environment: Implications from competitive ecology models. *Cancer Treat. Rept.*, **71**, 1093.

MICHELSON, S., GLICKSMAN, A.S. & LEITH, J.T. (1987*b*). Growth in solid heterogeneous human colon adenocarcinoma: Comparison of simple logistical models. *Cell Tissue Kinet.*, **20**, 343.

MICHELSON, S., LEITH, J.T. & GLICKSMAN, A.S. (1987*c*). A technique to analyze the emerging zonality of heterogeneous solid tumours. *Cell Tissue Kinet.*, **20**, 499.

MICHELSON, S., MILLER, B. GLICKSMAN, A.S. & 1 other (1987*a*). Tumor microecology and competitive interactions. *J. Theor. Biol.*, **128**, 233.

MICHELSON, S. & LEITH, J.T. (1988). Unexpected equilibria resulting from differing growth rates of subpopulations within heterogeneous tumors. *Math. Biosci.*, **91**, 119.

MILAS, L., ITO, H., HUNTER, N. & 2 others (1986). Retardation of tumor growth in mice caused by radiation-induced injury to tumor bed stroma: Dependency on tumor type. *Cancer Res.*, **46**, 723.

MILLER, B.E., MILLER, F.R., WILBURN, D.J. & 1 other (1987). Analysis of tumour cell composition in tumours composed of paired mixtures of mammary tumour cell lines. *Br. J. Cancer*, **56**, 561.

PETERS, L.J., BROCK, W.A., JOHNSON, T. & 3 others (1986). Potential methods for predicting tumor radiocurability. *Int. J. Radiat. Oncol. Biol. Phys.*, **12**, 459.

SIEMANN, D.W., LORD, E.M., KENG, P.C. & 1 other (1981). Cell subpopulations dispersed from solid tumours and separated by centrifugal elutriation. *Br. J. Cancer*, **44**, 100.

STEEL, G.G. (1988). The search for therapeutic gain in the combination of radiotherapy and chemotherapy. *Radiother. Oncol.*, **11**, 31.

TROTT, K.R. & KUMMEMEHR, J. (1983). Split dose recovery of a mouse tumour and stroma during fractionated irradiation. *Br. J. Radiol.*, **55**, 841.

URANO, M. & SUIT, H.D. (1970). Experimental evaluation of tumor bed effect of $C_3H$ mouse mammary carcinoma and for $C_3H$ fibrosarcoma. *Radiat. Res.*, **45**, 41.

VOGLER, H. & BECK-BORNHOLDT, H.-P. (1988). Radiotherapy of the rhabdomyosarcoma R1H of the rat: Kinetics of cellular inactivation by fractionated irradiation. *Int. J. Radiat. Oncol. Biol. Phys.*, **14**, 317.

WALLEN, C.A., MICHAELSON, S.M. & WHEELER, K.T. (1981). Influence of location within a tumor on cell survival as measured by a clonogenic assay. *Cancer Res.*, **41**, 1981.